# Simultaneous silencing Aurora-A and UHRF1 inhibits colorectal cancer cell growth through regulating expression of DNMT1 and STAT1

**DOI:** 10.7150/ijms.61969

**Published:** 2021-08-05

**Authors:** Jing Han, Xin Chen, Jiawei Xu, Laili Chu, Rongqing Li, Na Sun, Zhen Jiang, Hongyang Liu, Xing Ge, Junnian Zheng, Jing Yang, Takayuki Ikezoe

**Affiliations:** 1 Jiangsu Province Key Laboratory of Immunity and Metabolism, Xuzhou Medical University, Xuzhou, 221004, Jiangsu, China.; 2 Department of Pathogenic Biology and Immunology, Xuzhou Medical University, Xuzhou, 221004, Jiangsu, China.; 3 Jiangsu Center for the Collaboration and Innovation of Cancer Biotherapy, Cancer Institute, Xuzhou Medical University, Xuzhou, 221004, Jiangsu, China.; 4 National Experimental Demonstration Center for Basic Medicine Education, Xuzhou Medical University, Xuzhou, 221004, Jiangsu, China.; 5 Department of Oncology, the first affiliated hospital, Xuzhou Medical University, Xuzhou, 221004, Jiangsu, China.; 6 The Department of Hematology, Fukushima Medical University, Fukushima, Japan.

**Keywords:** Aurora-A, UHRF1, STAT1, colorectal cancer cell

## Abstract

Aurora-A has attracted a great deal of interest as a potential therapeutic target for patients with CRC. However, the outcomes of inhibitors targeting Aurora-A are not as favorable as expected, and the basis behind the ineffectiveness remains unknown. Here, we found that signal transducer and activator of transcription 1 (STAT1) was highly expressed in colorectal cancer (CRC) xenograft mouse models that were resistant to alisertib, an Aurora-A inhibitor. Unexpectedly, we found that alisertib disrupted Aurora-A binding with ubiquitin-like with plant homeodomain and ring finger domain 1 (UHRF1), leading to UHRF1 mediated ubiquitination and degradation of DNA methyltransferase 1 (DNMT1), which in turn resulted in demethylation of CpG islands of *STAT1* promoter and STAT1 overexpression. Simultaneous silencing Aurora-A and UHRF1 prevented STAT1 overexpression and effectively inhibited CRC growth. Hence, concomitant targeting Aurora-A and UHRF1 can be a promising therapeutic strategy for CRC.

## Introduction

Due to the changes in lifestyle and dietary behaviors, colorectal cancer (CRC) is one of the most common malignancies with substantial morbidity throughout the world 1. Ongoing advancements in diagnostics and treatment have improved the relative survival of CRC patients. While, CRC is still the third leading cause of cancer-related deaths in the world 2. To decrease mortality rates, the development of new treatment strategies or targeted therapy approaches, based on the molecular features of the tumor, is urgently needed.

Since Aurora-A was critical to cell division, overexpression of Aurora-A resulted in centrosome amplification, chromosomal instability and transformation in mammalian cells 3. Elevated expression of Aurora-A was found in various cases of human carcinogenesis. In more than 50% of primary CRC, aberrantly high levels of Aurora-A were observed 4. Additionally, Aurora-A has been implicated as a predictive recurrence marker in stage III CRC lacking microsatellite instability 5, and was also closely correlated with poor prognosis 4, 6-8. Recently several studies have found that Aurora-A lead to cancer cells' resistance to X-ray radiation and chemotherapy 6, 7. Silencing of Aurora-A could attenuate the resistance of those cancer cells to radiation or chemotherapy. Pharmacological inhibition of Aurora-A induced senescence in HCT-116 xenografts *in vivo* 9 as well as apoptosis of CRC cells 10. Importantly, recent study indicated that Aurora-A was required for tumorigenicity and chemoresistance of colorectal cancer stem cells 11. Knockdown of Aurora-A suppressed the tumor growth in immunocompromised mice and the migratory capacity of colorectal cancer stem cells 11. All of these findings indicated that targeting of Aurora-A could be useful strategy treatment for the individuals with CRC.

Several pharmaceutical companies have developed Aurora-A inhibitors, and some of the inhibitors have been assessed in clinical trials. Alisertib (MLN8237), developed by Millennium pharmaceuticals Inc., is an oral selective Aurora-A inhibitor with a half maximal inhibitory concentration (IC50) value of 1 nM 12. In animal models and phase I clinical trials, it has been shown that alisertib crosses the blood brain barrier, specifically at concentrations < the maximum tolerated dose. Several clinical trials have demonstrated that alisertib is generally well-tolerated in patients with solid tumors 13-15. Additionally, a phase II clinical trial indicated that alisertib yielded promising progression-free survival 16. A recent phase II clinical trial of alisertib as a single agent in advanced solid tumors such as gastroesophageal adenocarcinoma, revealed that 9% of patients (4 out of 47 patients) with gastroesophageal adenocarcinoma partially responded to the treatment 17, suggesting that more studies are needed to better characterize the role of Aurora-A in therapeutics for individuals with cancers. In some of the clinical trials, alisertib has been tested in combination with other drugs such as irinotecan, itraconazole or others (NCT01923337, NCT02259010). Currently, alisertib is being tested with relapsed or refractory peripheral T-cell lymphoma (NCT01482962), which is the first Aurora-A inhibitor to enter a phase III trial.

Ongoing clinical trials utilizing Aurora-A inhibitor alone, or in combination with traditional chemotherapies, are expected to provide important clinical information for strategy treatments of targeting Aurora-A in cancers 18. Herein, it is imperative to explore the potential mechanisms involvement in regulating of response or resistance to the inhibitors, which could improve evaluation of the efficacy of these drugs and establish biomarker based strategies for enrollment into clinical trials 18. Based on the importance of Aurora-A in CRC, therefore, we utilized a HCT-8 xenograft mouse model of human CRC cells to investigate the molecular mechanisms that could be involvement in attenuation of the anti-tumor effect of Aurora-A inhibitors, and attempted to develop novel treatment strategies to overcome the observed issues.

## Materials and methods

### Cells

Human normal colon cells FHC cells and human colorectal cancer HCT116, HT-29, SW480, Lovo, SW620, Colo205, HCT-8 cells and human glioma U251 cell line were commercially obtained from the Chinese Academy of Sciences Committee on Type Culture Collection Cell Bank (SCST, Shanghai, China). CRC cell lines were maintained in Dulbecco's Modified Eagle's Medium (DMEM) containing 10% heat-inactivated FBS, 100 mg/L streptomycin and 100 U/mL penicillin, at 37 °C in a 5% CO_2_ environment. U251 cells were cultured in OriCell^TM^ U251 human glioma cell growth medium (Cyagen Biosciences, Suzhou, Jiangsu, China) supplemented with 10% heat-inactivated fetal bovine serum (FBS, Clark, Australia), 100 mg/L streptomycin and 100 U/mL penicillin, at 37 °C in a 5% CO_2_ environment. U251AR were established by culturing the parental cells with increasing concentrations of alisertib (from 1 to 30 nM) for 6 months.

### Reagents

Alisertib and fludarabine were provided by Selleckchem (Houston, TX, USA), dissolved in 100% dimethyl sulfoxide (DMSO) (VICMED, Xuzhou, Jiangsu, China) to a stock concentration of 10^-3^ M, and stored at -80 °C. The final concentration of DMSO in the culture medium did not exceed 0.1%. At this concentration, DMSO alone had no effect on the cells' viability. Control siRNA and an siRNA against signal transducer and activator of transcription 1 (STAT1) were purchased from Jima Biotechnology (Shanghai, China). siSTAT1 sense: 5'-GCUGGAUGAUCAAUAUAGUTT-3'; antisense: 5'-ACUAUAUUGAUCAUCCAGCTT-3'.

### Tissue cDNA microarray

The mRNA levels of Aurora A and UHRF1 were determined on a CRC cDNA microarray purchased from Shanghai Outdo Biotech Company (Shanghai, China). MecDNA-HColA095Su01 contains 15 para-carcinoma tissue cDNA and 80 CRC tissues cDNA with a single core, according to the manufacturer's instructions.

The primer sets for PCR are shown in Table 1.

### Plasmid and transfection

The pCMV-ubiquitin-3flag-GFP-DNMT1 (NM_001130823) and control vectors were obtained from (Genechem., Shanghai, China). Cells were transiently transfected with control siRNA or siRNA against STAT1 utilizing siLentFect lipid reagent (BIO-RAD, Texas, USA), according to the manufacturer's instructions. For ectopic expression, cells were transiently transfected with either control or pCMV-ubiquitin-3flag-GFP-DNMT1 vector suing jetPRIME (polyplus, France), according to the manufacturer's instructions. After 24 h, cells were harvested and used for further experiments.

### short hairpin (sh) RNA lentivirus and infection

The shRNA lentivirus used to target either Aurora A or/and UHRF1 were obtained from (Genechem., Shanghai, China). SW480 cells were infected with shcontrol, shAurora A, shUHRF1 or shAu/UHRF1 according to the manufacturer's instructions. shAurora A: (NM_001323303.2, exon 10, GCCAGGGACCTCATTTCAAGA); 5'-CCGGGCCAGGGACCTCATTTCAAGACTCGAGTCTTGAAATGAGGTCCCTGGCTTTTTG-3'; shUHRF1: (NM_001048201, exon 12-13: CATGCAGTATCCAGAAGGCTA); 5'-CCGGCATGCAGTATCCAGAAG GCTACTCGAGTAGCCTTCTGGATACTGCATGTTTTTG-3'. After 24 h, 1 mL culture medium was added into the wells. On the third day, infected cells were cultured in DMEM culture medium containing 10% FBS and 2 mg/mL puromycin (VICMED, Xuzhou, Jiangsu, China) for 3 days.

### 3-(4,5-dimethylthiazol-2-yl)-2,5-diphenyltetrazolium bromide (MTT) assays

MTT were performed as described previously 19. Briefly, cells (2 × 10^5^/mL) were cultured with various concentrations of the indicated reagents or a DMSO control vehicle (the final concentration of DMSO did not exceed 0.1%). After 48 h, cell viability was measured by an MTT assay as previously described 20. All experiments were performed in triplicate, and repeated at least twice.

### 5-bromo-2^'^-deoxyuridine (BrdU) incorporation assay

Cells were transiently transfected with control siRNA or siRNA against STAT1. After 24 h, the transfected cells were co-cultured with 30 µM (final concentration) BrdU, and cell proliferation was assessed by the BrdU incorporation assay, according to the manufacturer's instrument (KeyGen Biotech., Nanjing, China).

### Colony formation assay

7 × 10^2^ infected cells were cultured in six-well plate at 37 °C for 10-14 days, visible colonies were washed twice with PBS, fixed with 4% paraformaldehyde. The number of colonies was counted under microscope.

### Microarray and data analysis

Control-treated xenografts and alisertib-treated xenografts were assayed for changes in genome-wide expression by the Affymetrix GeneChip array (Genechem., Shanghai, China). Gene network and pathway analyses were conducted using the Ingenuity Pathway Analysis (IPA, Ingenuity® Systems). Functional analysis of the resistant cell lines was performed using IPA with a 2-fold-change criteria, and a *P* value of < 0.05.

### RNA isolation and real-time reverse transcription-polymerase chain reaction (RT-PCR)

RNA isolation and cDNA preparation were performed as described previously 21, 22. We measured the expression of 18S for normalization, as previously described 21. Real time RT-PCR was performed using Power SYBR Green PCR Master Mix (TaKaRa, Japan), as described previously 21. The primer sets for PCR are shown in Table 1. The PCR conditions for all the genes were as follows: initial activation at 95 °C for 10 minutes, followed by 40 cycles at 95 °C for 15 s, and at 60 °C for 60 s, and fluorescence determination at the melting temperature of the product for 20 s on an ABI PRISM 7500 (Applied Biosystems).

### Mutational analysis of *AURKA*

DNA isolation was performed according to the manufacturer's instructions. Sequence analysis was performed to examine whether the acquired mutations occurred in the *AURKA* gene. The primer sets for PCR are shown in supplementary Table 1. PCR was carried out in a MycyclerTM thermal cycler (BIO-RAD). The PCR products were purified (Promega) and sent for sequence analysis (GENEWIZ, Suzhou, Jiangsu, China).

### Immunoblotting

The effect of alisertib on Aurora A and the Jak/STATs signaling pathways was assessed by immunoblotting, as described previously 21, 23, using the following phospho-specific antibodies: p-Aurora A/B/C (Cell Signaling Technology, #2914), Aurora A (35C1) (Abcam, ab13824), p-Jak1 (T1022/1023) (Cell Signaling Technology, #3331), Jak1 (6G4) (Cell Signaling Technology, #3344), p-Jak2 (T1007/1008) (Cell Signaling Technology, #3776), Jak2 (D2E12) (Cell Signaling Technology, #3230), p-STAT1 (T701) (Cell Signaling Technology, #7649), STAT1 (D1K9Y) (Cell Signaling Technology, #14994), p-STAT2 (T690) (Cell Signaling Technology, #4441), STAT2 (D9J7L) (Cell Signaling Technology, #72604), PARP (46D11) (Cell Signaling Technology, #9532), DNMT3B (D7070) (Cell Signaling Technology, #67259), DNMT1 (Proteintech., 24206-1-AP), DNMT3A (Proteintech., 19366-1-AP), ubiquitin (Proteintech., 10201-1-AP), UHRF1(Proteintech., 21402-1-AP), flag (Abmart, M20008L) and β-actin (Proteintech.,66009-1-Ig).

### *In vivo* ubiquitination assay

Cells were treated with 10 μM of an MG132 proteasome inhibitor for 5 h. Then, cells were trypsinized and collected. One-fifth of each sample was lysed with a 0.1% NP-40 lysis buffer, as input controls. The remaining cells were boiled in a hot 2% SDS buffer containing a 1× protease inhibitor (Sigma), 1 mM phenylmethylsulfonyl fluoride (PMSF), 1 mM sodium orthovanadate (NaVO_4_), and 1 mM dithiothreitol (DTT), for 10 minutes. Then, the lysates were diluted using a 0.1% NP-40 lysis buffer containing protease inhibitors, and subjected to immunoprecipitation. Western blotting was performed to identify the ubiquitinated forms of DNMT1.

### Identifying the interacting candidates of Aurora A

Cell lysates from HCT-8 cells stably expressing either Flag or Aurora A-Flag were immunoprecipitated by using anti-Flag M2 agarose beads (Sigma), and separated via SDS-PAGE. Liquid chromatography tandem mass spectrometry was performed on a LTQ-Orbitrap instrument (Thermo Electron Corporation). The data were analyzed using the IPI databases for human version 3.35 with BioWorks 3.2 software (Thermo Electron Corporation).

### Apoptosis assay

Cells were incubated with the indicated compounds for 24 or 48 h, and subsequently subjected to FACSCalibur to measure apoptosis using the annexin V-FITC apoptosis detection kit (KeyGen Biotech.), according to the manufacturer's instructions.

### Enzyme-linked immunosorbent assay (ELISA)

The serum of the xenografted mice was collected every week and the levels of IFN-α (Liankebio., Hangzhou, Zhejiang, China) and IFN-γ (Invitrogen) were examined utilizing an ELISA kit, according to the manufacturer's protocol.

### IF

Cells were plated at a density of 1 × 10^5^ cells/mL in glass-bottomed cell culture dishes (Nest, Wuxi, Jiangsu, China), fixed with 4% formaldehyde for 10 min, followed by 0.2% Triton X-100 for 5 min at 4 °C. After which, Cells were incubated with anti-STAT1 antibody at a dilution of 1:400. The following day, cells were incubated with the Alexa fluor-488-conjugated affinipure goat anti-rabbit (VICMED), followed by nuclear visualization staining with 4',6-diamidino-2-phenylindole (DAPI) (Invitrogen). Images were captured using a Zeiss LSM880 confocal microscope.

### Tumor tissue immunofluorescence staining

Tumor tissue immunofluorescence staining using PANO 3-plex IHC kit (Panovue, Beijing, China). Anti-STAT1 antibody was sequentially applied, followed by horseradish peroxidase-conjugated secondary antibody incubation and tyramide signal amplification. Nuclei were stained with DAPI after all the human antigens had been labelled.

### IHC

Xenografted tumors were fixed in 4% formaldehyde, and embedded in paraffin. Sections with a thickness of 4 µm were stained with hematoxylin-eosin (Beyotime Biotechnology, Nantong, Jiangsu, China). STAT1 expression was assessed using IHC as described previously 24. Briefly, the slides were pre-treated for antigen retrieval, followed by incubation with a 50:50 solution of 3% hydrogen peroxide and methanol for 20 min to block endogenous peroxidase and nonspecific background. The slides were incubated with anti-STAT1 antibody at a dilution of 1:400 for overnight at 4 °C. The following day, the slides were incubated with a biotinylated secondary antibody for 30 min, followed by incubation with avidin-biotin complex for 30 min. The slides were developed with 3,3-diaminobenzidine (DAB kit, Zsbio., Beijing, China). All the slides were lightly counterstained with hematoxylin for 30 s before dehydration and mounting.

### Methylation analysis by methylation-specific PCR and Bisulfite sequencing PCR

One microgram of DNA, isolated from either the control or alisertib-treated tumors, was used for bisulfite treatment using the EZ DNA Methylation kit (Zymo Research, Orange, CA). Two primer sets were used to amplify the promoter region of the *STAT1* gene that incorporated several CpG sites, one specific to the un-methylated sequence (STAT1-U, 204 bp) and the other to the methylated sequence (STAT1-M, 204 bp). The primer sets for PCR are shown in Table 2. Amplification was performed in a MycyclerTM thermal cycler (Thermo, USA) at 95 °C for 10 min, and cycled at 95 °C for 10 s, 52 °C for 30 s and 72 °C for 40 s (40 cycles), followed by a 10-min extension at 72 °C. Bisulfite sequencing analysis was performed as described previously 25.

### Alisertib-treated HCT-8 xenografts model

Five-week-old nu/nu athymic nude mice purchased from Vital River (Beijing, China) were injected subcutaneously with 1 × 10^6^ HCT-8 cells with the same volume of Matrigel (BD Biosciences). When the tumor volume reached 50 mm^3^, mice were randomized to receive either a drug diluent alone (control) or alisertib (20 mg/kg) orally, for 14 consecutive days, followed by 7 days off the drug. The intermittent drug cycle was repeated thrice. Tumor size was measured thrice a week using calipers. To further propagate the alisertib-treated tumors, tumors were removed (tumors #7 and #9), cells were cultured for 1 week, and reinjected subcutaneously into immunodeficient mice. When tumors were visible, the mice received either a drug diluent or alisertib (20 mg/kg) orally, for 14 consecutive days. To estimate fludarabine's ability in inhibiting the growth of xenografts, fludarabine (100 mg/kg) was given to the mice through an intraperitoneal injection eight times over 2 weeks of treatment. Tumor sizes were calculated using the formula: 4/3π × (a/2) × (b/2) × (c/2), where “a” is the length, “b” is the width, and “c” is the height in millimeters. At the end of the experiment, animals were killed by CO_2_ asphyxiation, and tumor weights were measured after careful resection. Tumor tissue was collected for analysis.

### Generation of conditional *AURKA* knockout mice

Conditional *AURKA* mice described as previously 26. Villin-Cre mice [B6.SJL-Tg(Vil-cre)997Gum/J] were intercrossed with mice carrying *loxP*-flanked *AURKA* alleles (*AURKA^f/f^*). PCR was utilized to identify the conditional AURKA knockout using primers (p5 5'-GGTAAGTGGTCTTGGGTGCT-3'; p6 5'- TAGCCAACTCATCTCCTCTG-3') and Villin-Cre (F 5'- TTTCCCGCAGAACCTGAAGA-3'; R 5'-GGTGCTAACCAGCGTTTTCGT-3') alleles. Mice were bred and maintained strictly under protocols approved by the Institutional Animal Care and Use Committee at Xuzhou Medical University.

### Statistical analysis

To compare the two groups, a Student's t-test was used. All statistical analyses were carried out using SPSS software (Version 18; SPSS, Tokyo, Japan), and the results were significant when the *P*-value was < 0.05, and highly significant when the* P*-value was < 0.01.

## Results

### Alisertib-treated xenograft models

**Figure 1:**
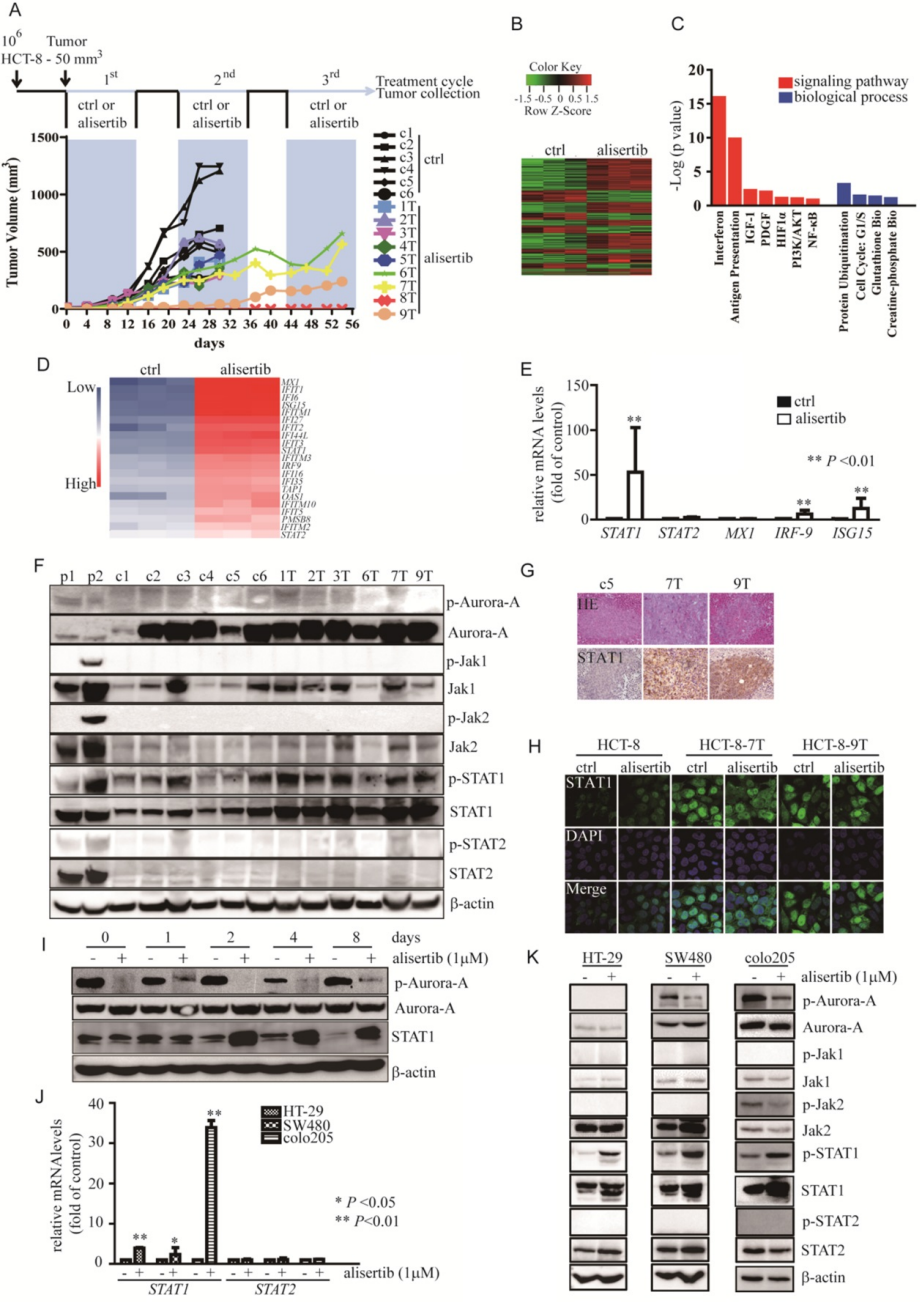
** Alisertib-treated xenograft models. (A)** Approximately 10^6^ HCT-8 cells were injected subcutaneously into the flanks of Balb/c nude mice. When tumors reached a volume of 50 mm^3^, mice were treated with control (n = 5, in black) or alisertib (n = 9, in color). After 14 days of treatment, drug administration was stopped for 7 days. The intermittent drug cycle was repeated three times. **(B)** The genes are shaded with green, black or red in the heat map to indicate low, intermediate or high expression, respectively. **(C)** Functional annotation clustering of genes regulated by long-exposure to alisertib is shown. Red, signaling pathway. Blue, biological process.** (D)** Gene expression levels of the subsets of genes involved in interferon signaling pathway. Blue, low expression. Red, high expression. **(E)** Real time RT-PCR was employed to measure the levels of the selected genes. Results represent the mean ± SEM. The statistical significance was assessed by a non-parametric test. **, *P*<0.01.** (F)** Immunoblotting was employed to measure the levels of Aurora-A and Jaks/STATs pathway in either control-treated or alisertib-treated cancer cells. **(G)** HE and STAT1 staining were performed on paraffin sections of tumors derived from control-treated and alisertib-treated mice as indicated.** (H)** The expression of STAT1 in established sublines HCT-8-7T, HCT-8-9T and HCT-8 cells were examined by IF. **(I)** HCT-8 cells were treated with 1 μM alisertib for the indicated time points. Cells were harvested and subjected to Immunoblotting to measure the levels of STAT1. **(J)** Real time RT-PCR was employed to measure the levels of *STAT1* and *STAT2* genes. Results represent the mean ± SEM. The statistical significance was assessed by a paired t-test. *, *P*<0.05; **, *P*<0.01. **(K)** HT-29, SW480 and Colo205 cells were treated with 1 μM alisertib. After 3 days later, cells were harvested and subjected to Immunoblotting.

Previously conducted studies have shown that the outcomes of alisertib administration were not as good as expected. To fully elucidate the mechanisms involved in the attenuation of the anti-tumor effects of alisertib, we established alisertib-treated xenograft models using HCT-8 cells possessing a moderate growth rate and Aurora-A expression (Figure S1A). Balb/c nude mice with HCT-8-induced tumors were randomized to receive either a saline control (n = 6) or 20 mg/kg/day of alisertib (n = 9). After 14 days of consecutive treatment, drug administration was interrupted for 7 days, and this on/off drug treatment regimen was repeated thrice, as described previously 27. As shown in Figure 1A, all tumors grew continuously in the control-treated mice. However, the tumors were initially suppressed in the alisertib-treated mice, especially tumor #8. During treatment, three tumors‑‑#6, #7 and #9 (6T, 7T and 9T)--were found to continuously grow in the presence of alisertib (Figure 1A). The tumors were isolated from #7 and #9, signal cells were prepared (labelled as HCT-8-7T and HCT-8-9T, respectively), and were re-implanted into the mice. When these cells formed palpable tumors, the mice were treated with either the control or alisertib for 14 days. Alisertib could not regress tumor growth (Figure S1B). These observations suggested that these cells harbored some functions that attenuated the anti-tumor effects of alisertib.

### STAT1 was amplified and activated in alisertib-treated tumors

To investigate the mechanisms by which HCT-8-7T and HCT-8-9T cells acquired an alisertib-resistant phenotype, we performed whole genome sequencing of the *AURKA* gene to examine if the acquired mutations result in alisertib resistance. Unfortunately, no mutation was identified in the *AURKA* gene (data not shown). To identify the key mediators involved in the attenuation of the anti-tumor effects of alisertib, microarray analysis was utilized to analyze the gene expression profile affected by alisertib. Hierarchical clustering revealed a total of 402 upregulated genes and 194 downregulated genes, in comparison to control-treated xenografts (Figure 1B). The IPA showed that the exposure of HCT-8 xenografts to alisertib primarily activated the interferon (IFN) signaling pathway (Figure 1C). Function annotation revealed the genes in interferon signaling pathway (Figure 1D). To elucidate the effect of alisertib on the IFN signaling pathway, the levels of IFN-α and IFN-γ in the serum of alisertib-treated mice were assessed. Additionally, the mRNA levels of IFN-α, IFN-β, and IFN-γ in tumors and CRC cells treated with alisertib were also measured. We found that the mRNA levels of IFN-α, IFN-β, and IFN-γ were increased in the alisertib-treated tumors as compared with control-treated tumors. Additionally, the levels of IFN-α, IFN-β, and IFN-γ mRNA were increased in alisertib-treated SW480 cells. The mRNA levels of IFN-α and IFN-γ, but not IFN-β, were also elevated in alisertib-treated HCT-8 cells, although the levels of IFN-α and IFN-γ were only slightly increased in the serum of the alisertib-treated mice, compared to those of the normal Balb/c nude mice (n = 3, Figure S1C-S1E), indicating that the secreted IFN could bind to their receptors expressing on cancer cells. On the other hand, contrary to the mRNA expressions of STAT2 and MX-1, the IFN-specific response marker 28, the levels of STAT1 mRNA and IFN-stimulated gene 15 (ISG15) mRNA were dramatically upregulated, in the alisertib-treated tumors (Figure 1E). Additionally, as shown in Figure 1F, treatment murine RAW267.4 cells with either nocodazole (negative control 1, p1) or the combination of 3ng/mL IFN-γ and 100 U/mL IFN-α (positive control 2, p2), the phosphorylated forms of Aurora-A and Jaks/STATs were obviously increased, respectively. On the other hand, without modulation of the levels of either the active form or total amount of Jak1/2 and STAT2, the levels of the phosphorylated form of STAT1, in parallel with the total amount of STAT1, were noticeably upregulated in the alisertib-treated tumors. However, the levels of the phosphorylated form of Aurora-A were almost undetectable in these tumors, and the total amount of Aurora-A in the alisertib-treated tumors was almost identical to that of the control-treated tumors (Figure 1F). Furthermore, the expression of STAT1 was consistently upregulated in the alisertib-treated xenografts, as measured by immunohistochemistry (IHC) (Figure 1G). The expression of STAT1 in the established HCT-8-7T and HCT-8-9T sublines was also explored by immunofluorescence (IF) after the exposure of these cells to a high dosage of alisertib (10 μM) for 24 h. Conversely, with the undetectable levels of STAT1 in the HCT-8 cells, constitutive nuclear expression of STAT1 was observed in these subline cells, even after exposure of these cells to 10 μM alisertib (Figure 1H).

To investigate whether expression of STAT1 was also upregulated after a short exposure of the cancer cells to alisertib, we evaluated the time course and found that the levels of STAT1 were obviously induced as early as at 48 h (Figure 1I). Similarly, mRNA and protein levels of STAT1 were indeed upregulated in alisertib-treated CRC cells (Figures 1J and 1K).

To further confirm if silencing of Aurora-A by genetic approaches contributed to an increase in the expressions of STAT1, we utilized conditional Aurora-A knockout mice established recently 26. Lethal phenotype was observed after intercrossing *AURKA^flox/+^;VillinCre^+^*with* AURKA^flox/+^* (data not shown), we therefore examined the STAT1 levels in epithelial cells isolated from intestine and colon tissues of *AURKA^flox/+^* or *AURKA^flox/+^;VillinCre^+^* mice, respectively. As our expected, the levels of STAT1 mRNA and protein were elevated in epithelial cells from intestine and colon tissues of *AURKA^flox/+^;VillinCre^+^* mice, compared to that of *AURKA^flox/+^* mice (Figures S2A-S2E).

Due to the feature of alisertib passing through the blood-brain barrier (BBB) 29, we exposed of human U251 cells to alisertib, to assess the generality that inhibition of Aurora-A induced upregulation of STAT1 in glioma, and found that long-term exposure of U251 cells to alisertib also enhanced STAT1 expression along with alisertib resistance (Figures S3A-S3D). These observations, together with the lack of mutation in *AURKA*, implicated that the cancer cells harboring resistance to alisertib could be dependent on the aberrant amplification and activation of STAT1 rather than acquired mutations in the *AURKA* gene or induction of IFN products *in vivo*, which implicated that inhibition of Aurora-A could generally elevate STAT1 expression.

### Overexpression of STAT1 endowed cancer cells resistance to alisertib

**Figure 2:**
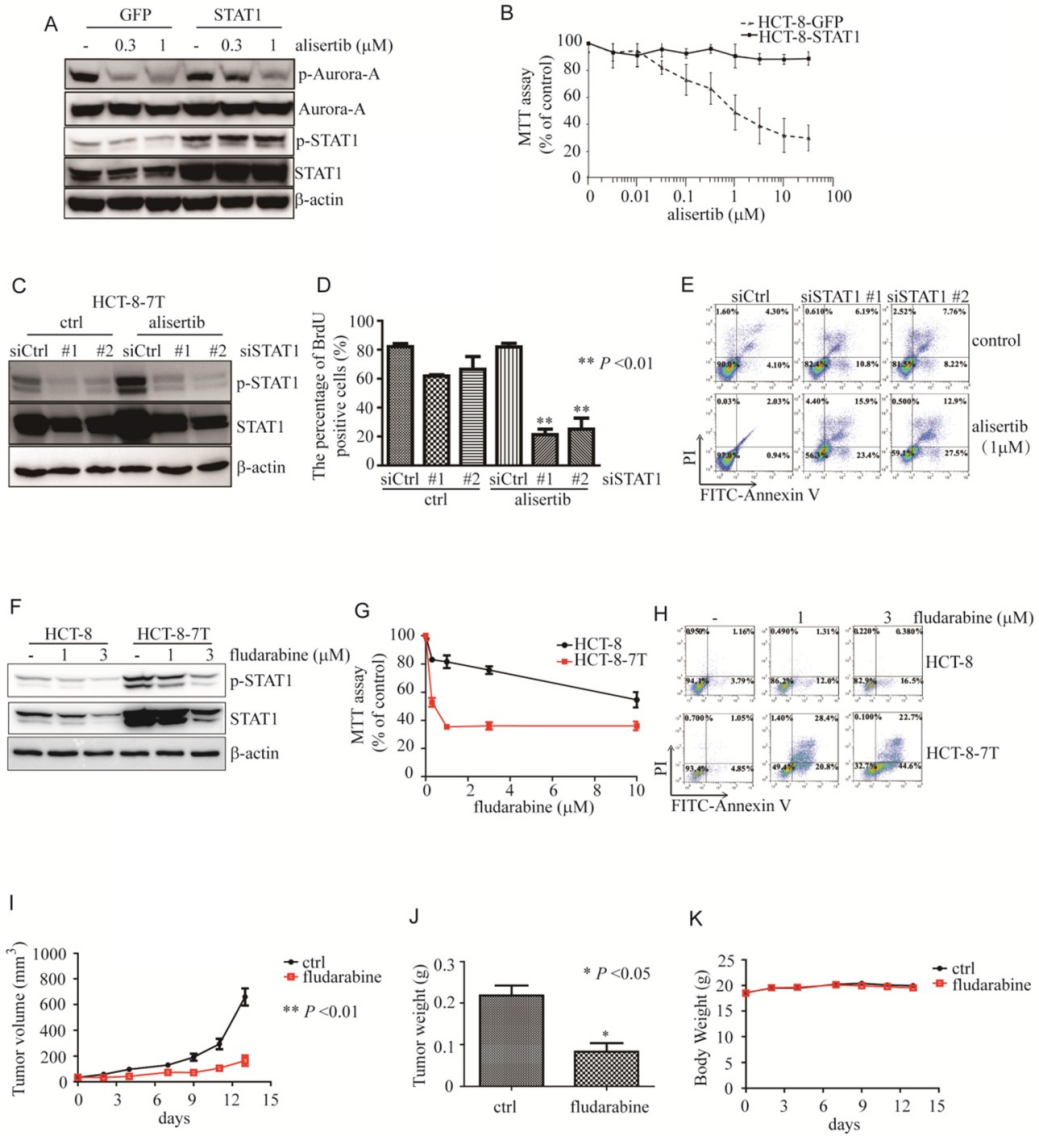
** Aberrant expression of STAT1 was critical for poor response to alisertib. (A)** Immunoblotting was performed to examine the levels of STAT1 in HCT-8 cells transiently infected with either control or STAT1 lentivirus.** (B)** Upon alisertib, the proliferation of above infected cells was measured by MTT assay. Results represent the mean ± SEM. **(C)** Immunoblotting was utilized to examine the levels of STAT1 in HCT-8-7T cells transiently transfected with either control or siSTAT1. **(D)** Upon alisertib, the proliferation of HCT-8-7T transiently transfected with either control or an siSTAT1 was examined by BrdU incorporation assay. Results represent the mean ± SEM. *, *P*<0.05; **, *P*<0.01.** (E)** Annexin staining and propidium iodide staining were analyzed by FACscan.** (F)** Immunolotting of protein lysates from HCT-8-7T cells in the presence of fludarabine. **(G)** The proliferation of HCT-8-7T cells in the presence of fludarabine was examined by MTT assay. Results represent the mean ± SEM. *, *P*<0.05; **, *P*<0.01.** (H)** Annexin staining and propidium iodide staining were analyzed by FACscan.** (I)** Upon fludarabine, tumor volumes of HCT-8-7T xenograft were measured every week. Results represent the mean ± SEM. **(J)** At end of the experiment, tumors were removed and weighed. Results represent the mean ± SEM. **(K)** Body weight of mice was measured twice a week during treatment.

To explore the role of STAT1 in alisertib-mediated growth inhibition, we overexpressed STAT1 and examined the anti-tumor ability of alisertib. As expected, this overexpression of STAT1 attenuated the alisertib-mediated growth suppression of the HCT-8 cells (Figures 2A and 2B). Also, forced expression of STAT1 endowed U251 cells resistance to alisertib (Figures S3E and S3F).

### Silencing of STAT1 by siRNA sensitized cancer cells to alisertib

To examine the function of the aberrant expression of STAT1 in the alisertib-treated cancer cells, we knocked down STAT1 and monitored the ability of alisertib to induce growth arrest and apoptosis in these alisertib-treated cells. As expected, two independent siRNAs against STAT1 effectively blocked both p-STAT1 and total amount of STAT1 expression in HCT-8-7T cells (Figure 2C). In addition, knockdown of STAT1 restored the sensitivity of HCT-8-7T cells to alisertib-mediated growth inhibition and apoptosis (Figures 2D and 2E). Similarly, knockdown of STAT1 also re-sensitized alisertib resistant U251 cells (U251AR) to alisertib-mediated growth inhibition and apoptosis (Figures S3G-S3I).

### Fludarabine induced growth arrest and apoptosis of alisertib-treated cancer cells *in vitro* and *in vivo*

To further investigate whether STAT1 was critical to the attenuation of the anti-tumor effects of alisertib, fludarabine, which has previously been shown to reduce mRNA levels and STAT1 phosphorylation, and downregulate the expression of STAT1, was utilized 30-32. We explored the potential of fludarabine to inhibit the proliferation of alisertib-treated cancer cells by MTT. Consistent with previous studies 30-32, fludarabine (1 or 3 μM) obviously reduced the levels of both p-STAT1 and STAT1 along with suppressing the proliferation of the HCT-8-7T cells in a dosage-dependent manner (Figures 2F and 2G). Whereas, the HCT-8 cells were relatively resistant to fludarabine (Figure 2G). On the contrary to HCT-8 cells, fludarabine dramatically induced apoptosis in the HCT-8-7T cells (Figure 2H).

We, next, evaluated the ability of fludarabine in inhibiting the growth of alisertib-resistant HCT-8-7T using an immunodeficient murine model. Tumor volumes and body weight were measured twice a week, and the tumor weights were determined at the time of necropsy. Fludarabine (100 mg/kg) markedly inhibited proliferation and tumor weights in the mice transplanted with alisertib-treated HCT-8-7T cells, compared to the control-treated mice (Figure 2I). The tumor weight in the mice that received fludarabine was significantly lower than that in the control-treated mice (Figure 2J). None of the mice showed signs of wasting (Figure 2K) or other toxicities (data not shown).

### Alisertib contributed to the epigenetic activation of STAT1 through the promotion of DNMT1 ubiquitination and degradation

**Figure 3:**
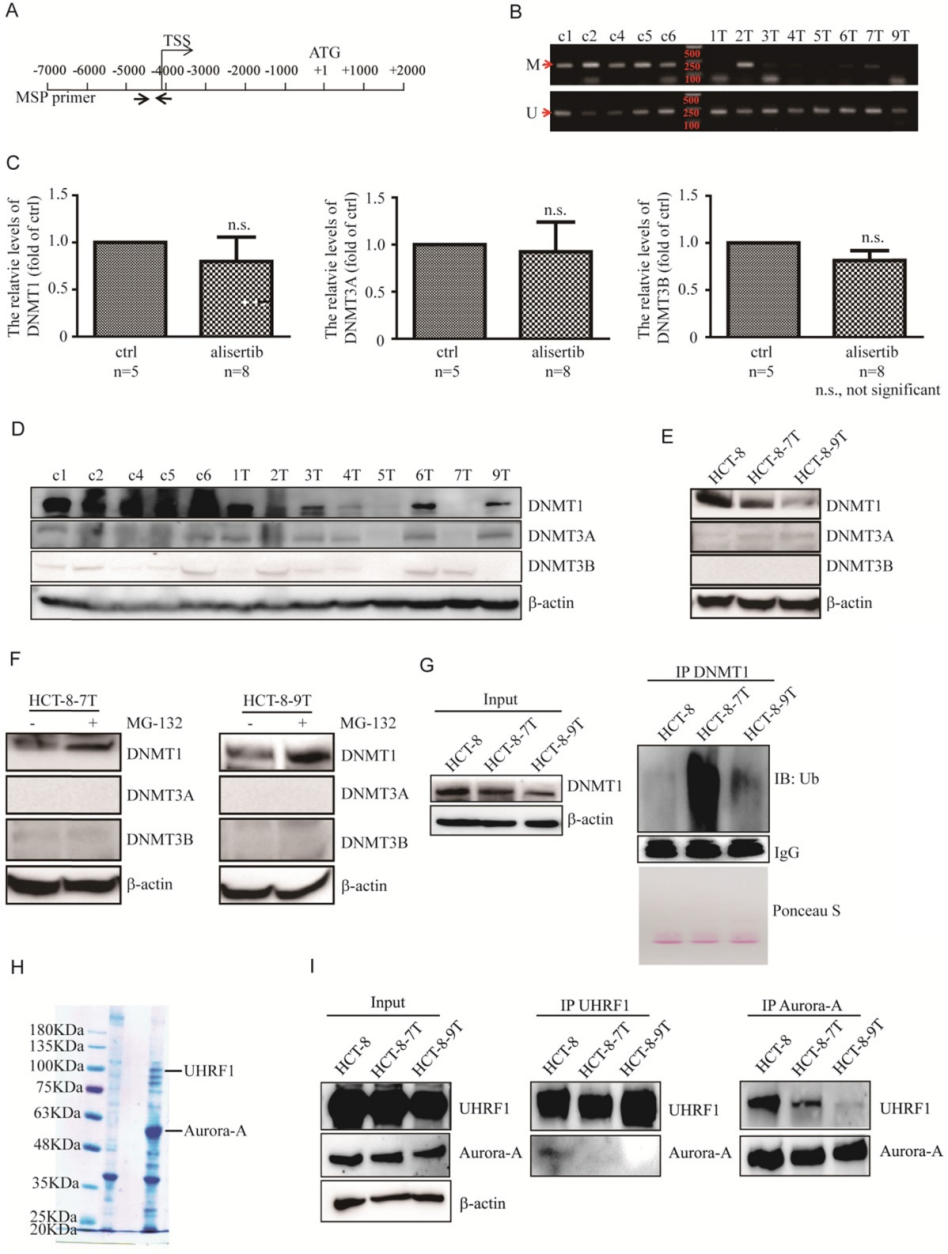
** Alisertib led to epigenetic activation of STAT1 via inducing DNMT1 degradation. (A)** Promoter region of the STAT1 gene and the location of PCR primers used in this study are shown. Numbering indicates the nucleotide position relative to the translation start codon (ATG), which is designated +1. **(B)** Un-methylation-specific and methylation-specific PCR products were amplified from control-treated and alisertib-treated HCT-8 xenograft tumors. U, unmethylated; M, methylated. **(C)** Real time RT-PCR was employed to measure the levels of DNMT1, DNMT3A and DNMT3B mRNA in the HCT-8 xenograft tumors. n.s., not significant. **(D-F)** Immunoblotting was employed to measure the levels of three members of DNMTs in HCT-8 xenograft tumors (D), HCT-8, HCT-8-7T and HCT-8-9T cells (E), HCT-8-7T or HCT-8-9T cells treated with or without MG-132 (10 μM) for 5 h (F). **(G)** HCT-8, HCT-8-7T and HCT-8-9T cells were treated with or without MG-132 (10 μM) for 5 h, and harvested. DNMT1 protein was IP with anti-DNMT1 antibody and analyzed by western blotting using an anti-ubiquitin antibody. **(H)** Cell lysates from HCT-8 cells stably expressing either Flag or Aurora-A-Flag were immunoprecipitated by using anti-Flag M2 agarose beads (Sigma), separated via SDS-PAGE and stained with CBB.** (I)** Cell lysates were harvested from indicated CRC cells, IP for either UHRF1 or Aurora-A, and immunoblotted with the indicated antibodies. Loading represents 5% of total cell lysate utilized for IP.

DNA methylation is critical to key biological processes, including differentiation and X chromosome inactivation 33. Failure to maintain proper DNA methylation leads to developmental disorders and cancer 33, 34. DNA hypermethylation of the promoters at the CpG islands usually hinders the binding of transcription factor, resulting in repressed gene transcription 35, 36. Due to elevated mRNA and protein expressions of STAT1 observed in alisertib-treated tumors without the activation of the IFN/Jaks signaling pathway, we therefore carefully examined the promoter region of the *STAT1* gene to confirm whether epigenetic modifications such as demethylation occurred in the alisertib-treated cells, utilizing methylation-specific PCR. The 204-bp product indicated a methylated STAT1 allele (Figure 3A). The CpG island in the promoter region of the *STAT1* gene demonstrated a lack of methylation in seven of eight of tumors isolated from the alisertib-treated HCT-8 xenografts, compared with zero of five tumors from the control-treated HCT-8 xenografts (Figure 3B). We further validated the methylation by bisulfite sequencing and found that the tumors isolated from alisertib-treated mice showed low methylation in the promoter region of STAT1 compared with the tumors removed from control-treated mice (Figure S4A).

So far, three active DNMTs have been identified in mammalian cells; we assessed their expression in the alisertib-treated HCT-8 xenograft tumors. Surprisingly, the expression of DNMT1 mRNA were almost identical, whereas the levels of DNMT1 protein were potently decreased in almost all of alisertib-treated HCT-8 xenograft tumors, compared to those in the control-treated HCT-8 xenograft tumors (Figures 3C and 3D). On the other hand, except tumors #5 (5T) and #7 (7T) that expressed almost undetectable levels of DNMT3A, no noticeable changes of both mRNA and protein levels of DNMT3A and DNMT3B were observed in the alisertib-treated HCT-8 xenograft tumors in comparison to those of the control-treated HCT-8 xenograft tumors (Figure 3D). Similarly, the expression of DNMT1 were obviously decreased HCT-8-7T and HCT-8-9T cells (Figure 3E). All of these observations indicated that DMNT1 could be involved in controlling the epigenetic activation of STAT1.

Our results eliminated the possibility that the downregulation of the transcriptional levels of DNMT1 could be the mechanism behind alisertib's reduction of DNMT1 levels. Thus, we sought to determine if alisertib promoted DNMT1 degradation via the ubiquitin-proteasome pathway. Treating the HCT-8-7T and HCT-8-9T cells with MG132 resulted in accumulation of DNMT1, but not DNMT3A or DNMT3B (Figure 3F). Furthermore, we performed polyubiquitination assays in the HCT-8, HCT-8-7T and HCT-8-9T cells. We treated these cells with MG132 to protect the polyubiquitinated DNMT1 species from degradation, and directly lysed the cells in a hot SDS lysis buffer to prevent DNMT1 deubiquitination during sample preparation. As shown in Figure 3G, high molecular weight smears, indicative of polyubiquitinated protein species, were observed in the alisertib-treated HCT-8-7T and HCT-8-9T cells (Figure 3G, lane 2 and 3), but not in their parental HCT-8 cells (Figure 3G, lane 1). These data indicated that alisertib induced the aberrant expression of STAT1, dependent on DNMT1 polyubiquitination and proteasomal degradation* in vivo*.

### Aurora-A and UHRF1 binding was interrupted in the presence of alisertib

We next identified the protein that interacted with Aurora-A and was also critical for Aurora-A-mediated DNMT1 degradation by Mass spectrometric analysis. The ubiquitin-like with plant homeodomain and ring finger domain 1 (UHRF1) tethered DNMT1 to replication forks to methylate synthesized DNA strands, and mediated the ubiquitination and degradation of DNMT1 34, 37, 38. We thus chose UHRF1 for further studies (Figure 3H). The interaction between Aurora-A and UHRF1 was validated via immunoprecipitation (IP) of endogenous proteins (Figure 3I). Interestingly, the IP of UHRF1 pulled down a significant amount of Aurora-A in the HCT-8 cell lysates (Figure 3I, middle panel, line 1). In comparison, the same amount of UHRF1 could not pull down the Aurora-A in the alisertib-treated tumors (Figure 3I, middle panel, line 2 and 3). Similarly, the IP of Aurora-A pulled down much more UHRF1 from the HCT-8 cell lysates than from the alisertib-treated subline cells' lysates (Figure 3I, right panel). Aurora-A-UHRF1 bindings were also abrogated in U251AR cells and SW480 cells after exposed to alisertib (Figures S4B and S4C).

To further explore whether alisertib facilitates UHRF1 binding to DNMT1, we treated the alisertib-resistant CRC cells with MG-132 for 5 h. Unfortunately, the IP of UHRF1 did not pull down DNMT1 *in vivo* (data not shown), perhaps as the UHRF1 antibody interferes with binding. To confirm if UHRF1 was critical for degradation of DNMT1 in alisertib-resistant CRC cells, we examined the expression of DNMT1 after knockdown UHRF1. Expected, the levels of DNMT1 were potently increased in parallel with a decrease in the levels of STAT1 (Figures S5A and S5B). To further test if DNMT1 was indeed involved in regulating STAT1 expression, we ectopically overexpressed DNMT1 in alisertib-resistant CRC cells and found that the levels of p-STAT1 and total amount of STAT1 were dramatically suppressed in DNMT1 expressing CRC cells, compared with that of control vector expressing CRC cells (Figures S5C and S5D). Furthermore, ectopic expression of DNMT1 restored the sensitivity of CRC cells to alisertib (Figures S5E and S5F). Together, these data demonstrated that UHRF1 interacted with Aurora-A* in vivo*; and alisertib promoted UHRF1-mediated DNMT1 degradation at least partially through the release of UHRF1 from Aurora-A binding.

### Simultaneous knockdown Aurora-A and UHRF1 inhibited proliferation of CRC cells

**Figure 4:**
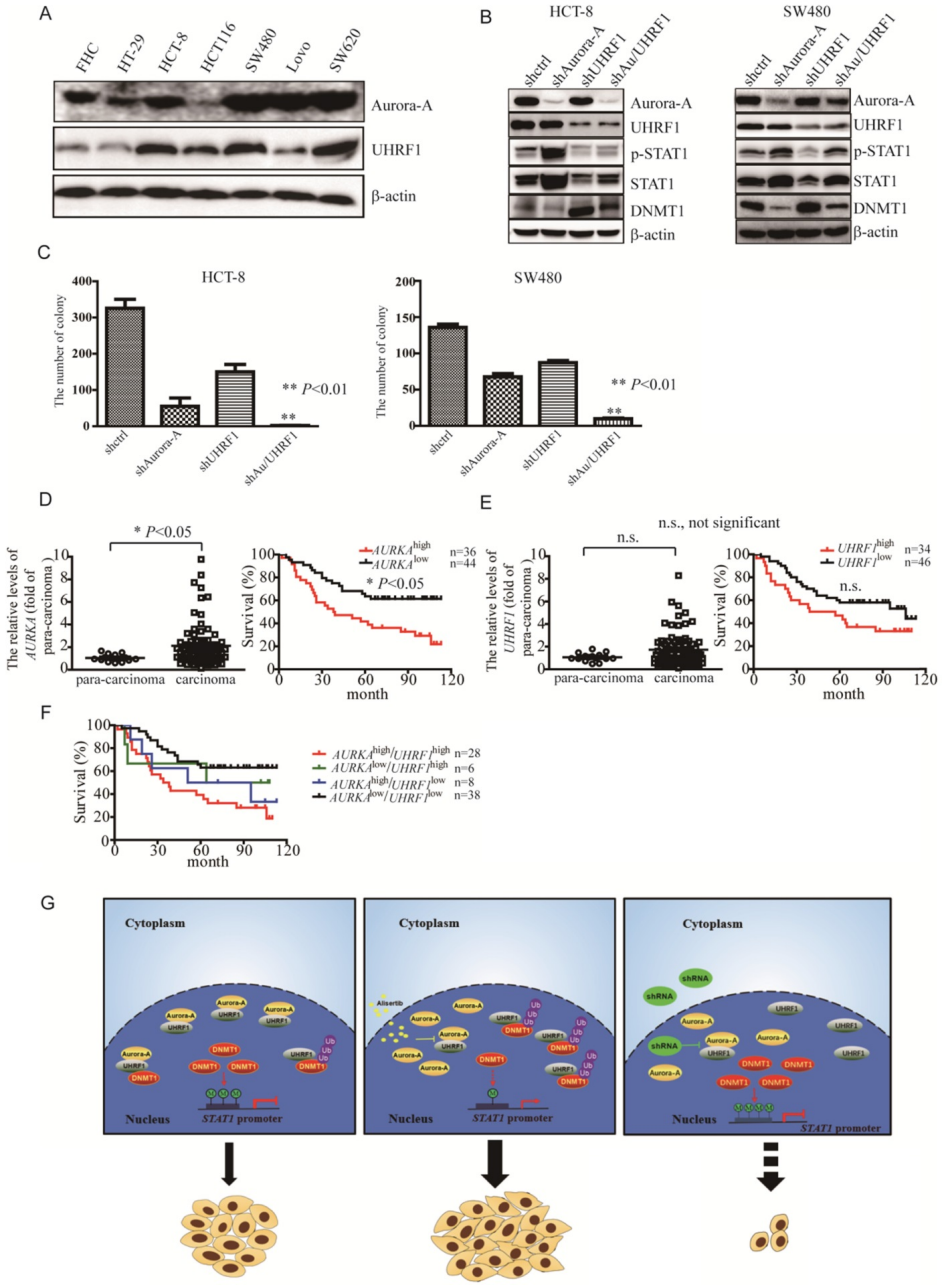
** Simultaneous targeting of Aurora-A and UHRF1 contributed to growth inhibition. (A-B)** Immunoblotting was employed in indicated CRC cells (A), HCT-8 or SW480 cells infected with indicated lentivirus (B).** (C)** The number of colonies was counted under microscope. Results represent the mean ± SEM. **, *P*<0.01. **(D)** Real time RT-PCR was employed to measure the levels of Aurora-A mRNA by tissue cDNA microarray (Left panel). Results represent the mean ± SEM. The statistical significance was assessed by a paired t-test. *, *P*<0.05. Kaplan-Meier analysis (Right panel) was performed and log-rank test used to make statistical comparisons. **(E)** Real time RT-PCR was employed to measure the levels of UHRF1 mRNA by tissue cDNA microarray (Left panel). Results represent the mean ± SEM. The statistical significance was assessed by a paired t-test. n.s., not significant. Kaplan-Meier analysis (Right panel) was performed and log-rank test used to make statistical comparisons. **(F)** Kaplan-Meier analysis was performed and log-rank test used to make statistical comparisons. **(G)** Signal pathways in control/untreated and alisertib-treated cancer cells. In control/untreated cancer cells, Aurora-A interacted with UHRF1, protecting DNMT1 from UHRF1-mediated degradation. DNMT1 primarily localized in nuclear and mediated methylation forms of STAT1, leading to suppressing its transcriptional activity and down-regulating the expression of its down-stream targets genes (left panel). Exposure of cancer cells to alisertib led to UHRF1 released from Aurora-A binding, resulting in DNMT1 degradation through ubiquitination and proteasomal pathway, which caused STAT1 demethylation and up-regulation its transcriptional activity, and subsequently led to resistance to alisertib (middle panel). Concomitant targeting of Aurora-A and UHRF1 inhibited proliferation in association with controlling STAT1 expression (right panel).

To further investigate if the levels of STAT1 mediated by Aurora-A/UHRF1 pathway is required for proliferation of CRC, we performed the colony formation assay after silencing of Aurora-A or/and UHRF1. Contrary to SW620 cells expressing higher levels of Aurora-A and UHRF1, HCT-8 as well as SW480 cells moderately expressed Aurora-A and UHRF1 (Figure 4A), which would be a better representative for CRC than those expressing Aurora-A either too low or too high. Therefore, we targeted Aurora-A or/and UHRF1 in HCT-8 and SW480 cells. Comparison to shcontrol lentivirus, shRNA lentiviral particle against Aurora-A effectively decreased Aurora-A expression (Figure 4B, HCT-8 panel, line 2). Nevertheless, the levels of STAT1 were dramatically increased in shAurora-A infected HCT-8 cells (Figure 4B, HCT-8 panel, line 2). ShRNA lentivirus against UHRF1 also effectively blocked the expression of UHRF1, compared with shcontrol lentivirus. Unexpectedly, the levels of Aurora-A were also slightly reduced in those shUHRF1 infected HCT-8 cells (Figure 4B, HCT-8 panel, line 3). As our expected, the levels of STAT1 were decreased in parallel with up-regulation of DNMT1 expression (Figure 4B, HCT-8 panel, line 3). Simultaneously targeted Aurora-A and UHRF1 rescued the levels of DNMT1 mediated by shRNA against Aurora-A, and effectively blocked STAT1 expression (Figure 4B, HCT-8 panel, line 4). Consistent results were also observed in SW480 cells after silencing of Aurora-A or/and UHRF1 (Figure 4B, SW480 panel). Furthermore, concomitant knockdown Aurora-A and UHRF1 potently inhibited the growth of HCT-8 and SW480 cells (Figure 4C). We also investigated the levels of Aurora-A and UHRF1 in individuals with CRC by tissue cDNA microarray. The cutoff values were examined by SPSS software. Individuals with higher *AURKA* gene expression had significant shorter survival (Figure 4D). It seemed that overexpression of *UHRF1* gene were associated with signs of poor clinical outcome (Figure 4E, *P* = 0.064). Moreover, it seemed that *AURKA*^high^/*UHRF1*^high^ expression predicted an inferior overall survival when compared with *AURKA*^low^/*UHRF1*^high^ or *AURKA*^low^/*UHRF1*^low^ expression (Figure 4F).

## Discussion

STAT1 is usually activated by IFN-γ, and closely related with the pro-apoptotic pathway 39. Nevertheless, under certain stresses, STAT1 was required for cell survival and drug resistance. The upregulation of *STAT1* is associated with docetaxel resistance in prostate cancer cells 40 and cisplatin resistance in ovarian cancer cells 41. Myeloma cells are endowed with acquired cross-resistance to doxorubicin and radiation when they have an over-expression of STAT1 mRNA and protein levels 42. Consistent with previously conducted studies, this study also demonstrated that exposure to alisertib contributed to cancer cells harboring relative resistance to it, in association with the aberrant expression of STAT1 mRNA and protein levels (Figure 1). The silencing of STAT1 by siRNAs significantly re-sensitized these cells to alisertib (Figures 2C-2E). Fludarabine potently inhibited the proliferation and induced apoptosis of the alisertib-treated cancer cells *in vitro* as well as* in vivo* (Figures 2F-2I). In comparison with HCT-8-7T cells, lower expression of STAT1 was observed in HCT-8 cells, which could lead to relative resistance to fludarabine (Figures 2F-2I). We observed the upregulated expression of STAT1 in the alisertib-treated cancer cells (Figure 1). Another study found that the blockade of Jaks by AG490 enhanced the sensitivity of cisplatin-resistant ovarian cancer cells to AMD473, but not cisplatin 41, highlighting the importance of an elevated expression of STAT1 in the acquisition of the “off-target effects of alisertib”. Over-expressed histone deacetylase 4 (HDAC4) interacted with STAT1, decreasing the levels of STAT1 acetylation and promoting STAT1 nuclear localization, thereby leading to cisplatin resistance in ovarian cancers 39. However, the levels of HDAC4 mRNA in alisertib-treated cells were almost identical to those of their parental cells (data not shown).

We assumed that STAT1 could be subjected to lack of methylation in the acquired relative resistance to alisertib, and investigated if STAT1 was de-methylated in the promoter region of the *STAT1* gene. As expected, alisertib-treated mice showed low methylation in the promoter region of STAT1 compared with the tumors removed from control-treated mice (Figures 3B and S4A), indicating that exposure to alisertib could cause the lack of methylation of STAT1, resulting in a poor response to alisertib.

Similar to the findings in this study, it has been shown that the suppression of STAT1 methylation was also involved in the resistance of hepatitis B virus (HBV) to IFN-α 43. The epigenetic activation of the *STAT1* gene may be a universal event in various types of cancers, especially in the IFN-resistance of cancer cells or individuals with a poor response to alisertib. Future experiments could analyze the STAT1 expression as well as methylation status of the *STAT1* gene in samples isolated from patients who have poor response to alisertib therapy, and confirm the clinical relevancy of this study's observations.

As DNMT1, DNMT3A, and DNMT3B are involved in the maintenance of the methylation patterns of genes 36, we assessed their expression. Our results demonstrated that DNMT1, but not DNMT3A and DNMT3B, was degraded through the ubiquitin proteasome pathway (Figures 3C-3G). Other studies have demonstrated that AKT phosphorylated the Ser143 residue of DNMT1 and subsequently rendered DNMT1 resistant to proteasomal degradation 36, 44. However, we found that the levels of both the phosphorylated forms and the total amount of AKT in the alisertib-treated cancer cells were identical to those of their parental cells (data not shown). A number of studies have shown that UHRF1 possesses E3 ubiquitin ligase activity in its RING finger domain and interacts with DNMT1 34, 37, 38. Upon Aurora-A inhibitor, such as alisertib, the levels of STAT1 were dramatically enhanced (Figure 1). Similarly, silencing of Aurora-A also induced upregulation of STAT1 (Figures 4B, S2B-S2F). On the other hand, silencing of UHRF1 potently decreased the levels of both p-STAT1 and total amount of STAT1 in association with an increase in the levels of DNMT1 (Figures 4B, S5A and S5B), indicating that upon inhibition of Aurora-A, UHRF1 could be released through Aurora-A binding, which, in turn, promoted the ubiquitination and degradation of DNMT1, and induced epigenetic activation of STAT1, although the UHRF1-DNMT1 binding affinity could not be examined *in vivo*. While, simultaneously targeted Aurora-A and UHRF1 effectively blocked STAT1 expression in associated with suppression the proliferation of HCT-8 and SW480 cells (Figures 4B and 4C). Silencing UHRF1 could contribute to DNMT1 stabilization, leading to demethylation STAT1 promoter thereby decreasing the expression of STAT1, which could sensitize those resistant cells to alisertib. Nevertheless, based on the observations that UHRF1 play an important role in controlling cell cycle 45, 46, we cannot exclude the possibility that the additive effect of simultaneous knockdown of Aurora-A and UHRF1 on cell growth may be independent of DNMT1.

In summary, the exposure of cancer cells to alisertib induced the upregulation of STAT1 in association with the lack of methylation on the promoter region of this gene. The epigenetic activation of STAT1, in parallel with the release of UHRF1 from Aurora-A binding, and promotion of DNMT1 ubiquitination and degradation, could contribute to the poor response to alisertib, at least, practically (Figure 4G). Concomitant administration of an Aurora-A inhibitor and an UHRF1 inhibitor could provide a novel strategy to overcome drug insensitivity in CRC (Figure 4G).

## Supplementary Material

Supplementary figures and tables.

## Figures and Tables

**Table 1:** RT-PCR primers

Gene	Direction	Primer
*AURKA*	Forward	5'-CCACCTTCGGCATCCTAATA-3'
	Reverse	5'-TCCAAGTGGTGCATATTCCA-3'
*UHRF1*	Forward	5'-TCAAGGGTGGCAAGAATAGC-3'
	Reverse	5'-TTCTCGGGCCAGTATTTCAC-3'
*STAT1*	Forward	5'-TTCAGAGCTCGTTTGTGGTG-3'
	Reverse	5'-TGAACTGGACCCCTGTCTTC-3'
*STAT2*	Forward	5'-AACCGTACACGAAGGAGGTG-3'
	Reverse	5'-GATTCGGGGATAGAGGAAGC-3'
*MX1*	Forward	5'-ACAGGACCATCGGAATCTTG-3'
	Reverse	5'-CTTCAGGTGGAACACGAGGT-3'
*IRF-9*	Forward	5'-GTACCATCAAAGCGACAGCA-3'
	Reverse	5'-CAGAGGGACTGAGTGTGCAG-3'
*ISG15*	Forward	5'-GACCTGACGGTGAAGATGCT-3'
	Reverse	5'-CGATCTTCTGGGTGATCTGC-3'
*DNMT1*	Forward	5'-GCAAGAAGTGAAGCCCGTAG-3'
	Reverse	5'-TGAACGCTTAGCCTCTCCAT-3'
*DNMT3A*	Forward	5'-GCCAACGATTGCTAGACTGG-3'
	Reverse	5'-ATGGCACGTGGGAGAGTAAG-3'
*DNMT3B*	Forward	5'-CACGCAACCAGAGAACAAGA-3'
	Reverse	5'-CTCGGCTCTGATCTTCATCC-3'
*18S*	Forward	5'-AAACGGCTACCACATCCAAG-3'
	Reverse	5'-CCTCCAATGGATCCTCGTTA-3'

**Table 2:** PCR primers used for methylation-specific PCR

Gene	Direction	Primer
*STAT1*-M	Forward	5'-GCGTTTTTTGGGTTTAGTAATAC-3'
	Reverse	5'-ATTACCACGCTAAAAACTAACGTT-3'
*STAT1*-U	Forward	5'-GTGTTTTTTGGGTTTAGTAATATGG-3'
	Reverse	5'-TTACCACACTAAAAACTAACATT-3'

## References

[1] Rawla P, Sunkara T, Barsouk A (2019). Epidemiology of colorectal cancer: incidence, mortality, survival, and risk factors. Przeglad gastroenterologiczny.

[2] Feng RM, Zong YN, Cao SM, Xu RH (2019). Current cancer situation in China: good or bad news from the 2018 Global Cancer Statistics?. Cancer Commun (Lond).

[3] Zhou H, Kuang J, Zhong L, Kuo WL, Gray JW, Sahin A (1998). Tumour amplified kinase STK15/BTAK induces centrosome amplification, aneuploidy and transformation. Nature genetics.

[4] Bischoff JR, Anderson L, Zhu Y, Mossie K, Ng L, Souza B (1998). A homologue of Drosophila aurora kinase is oncogenic and amplified in human colorectal cancers. The EMBO journal.

[5] Belt EJ, Brosens RP, Delis-van Diemen PM, Bril H, Tijssen M, van Essen DF (2012). Cell cycle proteins predict recurrence in stage II and III colon cancer. Annals of surgical oncology.

[6] Anand S, Penrhyn-Lowe S, Venkitaraman AR (2003). AURORA-A amplification overrides the mitotic spindle assembly checkpoint, inducing resistance to Taxol. Cancer cell.

[7] Xu J, Yue CF, Zhou WH, Qian YM, Zhang Y, Wang SW (2014). Aurora-A contributes to cisplatin resistance and lymphatic metastasis in non-small cell lung cancer and predicts poor prognosis. Journal of translational medicine.

[8] Isola JJ, Kallioniemi OP, Chu LW, Fuqua SA, Hilsenbeck SG, Osborne CK (1995). Genetic aberrations detected by comparative genomic hybridization predict outcome in node-negative breast cancer. The American journal of pathology.

[9] Huck JJ, Zhang M, McDonald A, Bowman D, Hoar KM, Stringer B (2010). MLN8054, an inhibitor of Aurora A kinase, induces senescence in human tumor cells both *in vitro* and *in vivo*. Molecular cancer research: MCR.

[10] Kuratnik A, Senapati VE, Verma R, Mellone BG, Vella AT, Giardina C (2012). Acute sensitization of colon cancer cells to inflammatory cytokines by prophase arrest. Biochemical pharmacology.

[11] Cammareri P, Scopelliti A, Todaro M, Eterno V, Francescangeli F, Moyer MP (2010). Aurora-a is essential for the tumorigenic capacity and chemoresistance of colorectal cancer stem cells. Cancer research.

[12] Carvajal RD, Tse A, Schwartz GK (2006). Aurora kinases: new targets for cancer therapy. Clinical cancer research: an official journal of the American Association for Cancer Research.

[13] Cervantes A, Elez E, Roda D, Ecsedy J, Macarulla T, Venkatakrishnan K (2012). Phase I pharmacokinetic/pharmacodynamic study of MLN8237, an investigational, oral, selective aurora a kinase inhibitor, in patients with advanced solid tumors. Clinical cancer research: an official journal of the American Association for Cancer Research.

[14] Dees EC, Cohen RB, von Mehren M, Stinchcombe TE, Liu H, Venkatakrishnan K (2012). Phase I study of aurora A kinase inhibitor MLN8237 in advanced solid tumors: safety, pharmacokinetics, pharmacodynamics, and bioavailability of two oral formulations. Clinical cancer research: an official journal of the American Association for Cancer Research.

[15] Falchook G, Kurzrock R, Gouw L, Hong D, McGregor KA, Zhou X (2014). Investigational Aurora A kinase inhibitor alisertib (MLN8237) as an enteric-coated tablet formulation in non-hematologic malignancies: phase 1 dose-escalation study. Investigational new drugs.

[16] Dickson MA, Mahoney MR, Tap WD, D'Angelo SP, Keohan ML, Van Tine BA (2016). Phase II study of MLN8237 (Alisertib) in advanced/metastatic sarcoma. Annals of oncology: official journal of the European Society for Medical Oncology.

[17] Melichar B, Adenis A, Lockhart AC, Bennouna J, Dees EC, Kayaleh O (2015). Safety and activity of alisertib, an investigational aurora kinase A inhibitor, in patients with breast cancer, small-cell lung cancer, non-small-cell lung cancer, head and neck squamous-cell carcinoma, and gastro-oesophageal adenocarcinoma: a five-arm phase 2 study. The Lancet Oncology.

[18] Katsha A, Belkhiri A, Goff L, El-Rifai W (2015). Aurora kinase A in gastrointestinal cancers: time to target. Molecular cancer.

[19] Li Y, Baiyang L, Leran B, Zhen W, Yandong X, Baixiang D (2017). Reduction-responsive PEtOz-SS-PCL micelle with tailored size to overcome blood-brain barrier and enhance doxorubicin antiglioma effect. Drug delivery.

[20] Nishioka C, Ikezoe T, Yang J, Koeffler HP, Taguchi H (2007). Fludarabine induces apoptosis of human T-cell leukemia virus type 1-infected T cells via inhibition of the nuclear factor-kappaB signal pathway. Leukemia.

[21] Yang J, Ikezoe T, Nishioka C, Tasaka T, Taniguchi A, Kuwayama Y (2007). AZD1152, a novel and selective aurora B kinase inhibitor, induces growth arrest, apoptosis, and sensitization for tubulin depolymerizing agent or topoisomerase II inhibitor in human acute leukemia cells *in vitro* and *in vivo*. Blood.

[22] Wang H, Zhang R, Zhu Y, Teng T, Cheng Y, Chowdhury A (2019). Microsomal prostaglandin E synthase 2 deficiency is resistant to acetaminophen-induced liver injury. Archives of toxicology.

[23] Lu J, Wang H, Zhang R, Wan Z, Gao H, Cai J (2019). Effects of Photoperiod on Acetaminophen-Induced Hepatotoxicity in Mice. Digestive diseases and sciences.

[24] Ding Y, Shi W, Xie G, Yu A, Wang Q, Zhang Z (2015). CX3CR1 Mediates Nicotine Withdrawal-Induced Hyperalgesia via Microglial P38 MAPK Signaling. Neurochemical research.

[25] McGough JM, Yang D, Huang S, Georgi D, Hewitt SM, Rocken C (2008). DNA methylation represses IFN-gamma-induced and signal transducer and activator of transcription 1-mediated IFN regulatory factor 8 activation in colon carcinoma cells. Molecular cancer research: MCR.

[26] Han J, Jiang Z, Wang C, Chen X, Li R, Sun N (2020). Inhibition of Aurora-A Promotes CD8(+) T-Cell Infiltration by Mediating IL10 Production in Cancer Cells. Molecular cancer research: MCR.

[27] Pirazzoli V, Nebhan C, Song X, Wurtz A, Walther Z, Cai G (2014). Acquired resistance of EGFR-mutant lung adenocarcinomas to afatinib plus cetuximab is associated with activation of mTORC1. Cell reports.

[28] Muller U, Steinhoff U, Reis LF, Hemmi S, Pavlovic J, Zinkernagel RM (1994). Functional role of type I and type II interferons in antiviral defense. Science.

[29] Hill RM, Kuijper S, Lindsey JC, Petrie K, Schwalbe EC, Barker K (2015). Combined MYC and P53 defects emerge at medulloblastoma relapse and define rapidly progressive, therapeutically targetable disease. Cancer cell.

[30] Frank DA, Mahajan S, Ritz J (1999). Fludarabine-induced immunosuppression is associated with inhibition of STAT1 signaling. Nature medicine.

[31] Terui K, Haga S, Enosawa S, Ohnuma N, Ozaki M (2004). Hypoxia/re-oxygenation-induced, redox-dependent activation of STAT1 (signal transducer and activator of transcription 1) confers resistance to apoptotic cell death via hsp70 induction. The Biochemical journal.

[32] Fagard R, Mouas H, Dusanter-Fourt I, Devillers C, Bissieres P, Martin A (2002). Resistance to fludarabine-induced apoptosis in Epstein-Barr virus infected B cells. Oncogene.

[33] Bird A (2007). Perceptions of epigenetics. Nature.

[34] Du Z, Song J, Wang Y, Zhao Y, Guda K, Yang S (2010). DNMT1 stability is regulated by proteins coordinating deubiquitination and acetylation-driven ubiquitination. Science signaling.

[35] Karpf AR, Jones DA (2002). Reactivating the expression of methylation silenced genes in human cancer. Oncogene.

[36] Agarwal S, Amin KS, Jagadeesh S, Baishay G, Rao PG, Barua NC (2013). Mahanine restores RASSF1A expression by down-regulating DNMT1 and DNMT3B in prostate cancer cells. Molecular cancer.

[37] Harrison JS, Cornett EM, Goldfarb D, DaRosa PA, Li ZM, Yan F (2016). Hemi-methylated DNA regulates DNA methylation inheritance through allosteric activation of H3 ubiquitylation by UHRF1. eLife.

[38] Liu X, Gao Q, Li P, Zhao Q, Zhang J, Li J (2013). UHRF1 targets DNMT1 for DNA methylation through cooperative binding of hemi-methylated DNA and methylated H3K9. Nature communications.

[39] Stronach EA, Alfraidi A, Rama N, Datler C, Studd JB, Agarwal R (2011). HDAC4-regulated STAT1 activation mediates platinum resistance in ovarian cancer. Cancer research.

[40] Patterson SG, Wei S, Chen X, Sallman DA, Gilvary DL, Zhong B (2006). Novel role of Stat1 in the development of docetaxel resistance in prostate tumor cells. Oncogene.

[41] Roberts D, Schick J, Conway S, Biade S, Laub PB, Stevenson JP (2005). Identification of genes associated with platinum drug sensitivity and resistance in human ovarian cancer cells. British journal of cancer.

[42] Fryknas M, Dhar S, Oberg F, Rickardson L, Rydaker M, Goransson H (2007). STAT1 signaling is associated with acquired crossresistance to doxorubicin and radiation in myeloma cell lines. International journal of cancer.

[43] Li J, Chen F, Zheng M, Zhu H, Zhao D, Liu W (2010). Inhibition of STAT1 methylation is involved in the resistance of hepatitis B virus to Interferon alpha. Antiviral research.

[44] Esteve PO, Chang Y, Samaranayake M, Upadhyay AK, Horton JR, Feehery GR (2011). A methylation and phosphorylation switch between an adjacent lysine and serine determines human DNMT1 stability. Nature structural & molecular biology.

[45] Avvakumov GV, Walker JR, Xue S, Li Y, Duan S, Bronner C (2008). Structural basis for recognition of hemi-methylated DNA by the SRA domain of human UHRF1. Nature.

[46] Hashimoto H, Horton JR, Zhang X, Bostick M, Jacobsen SE, Cheng X (2008). The SRA domain of UHRF1 flips 5-methylcytosine out of the DNA helix. Nature.

