# Supplementary material for: Simultaneous silencing Aurora-A and UHRF1 inhibits colorectal cancer cell growth through regulating expression of DNMT1 and STAT1
Source: Int J Med Sci. 2021 Aug 5;18(15):3437–51. doi: 10.7150/ijms.61969 (PMC8436113; doi:10.7150/ijms.61969)

Supplemental Figure legends

Figure S1. Alisertib-induced STAT1 expression was in an interferon/Janus kinases-independent manner, related to Figure 1.

(A) Immunoblotting was employed to measure the levels of Aurora-A in indicated CRC cells. β-actin served as the control. **(B)** Tumor growth of the transplants derived from alisertib-treated tumors #7 (left) and #9 (right). The alisertib-treated tumors were removed. Signal cells were prepared, grown in DMEM for 7 days and transplanted into nude mice. When these cells formed palpable tumors, mice were treated continuously with either control (in black) or alisertib (in color). Transplants are labeled with the number of the original tumor they were derived from (#7 or 9), the letter "T," and a number. **(C)** Serum from normal Balb/c nude mice (n=3), control-treated or alisertib-treated mice were collected during the treatment, and the levels of IFN-α and IFN-γ were examined by ELISA. Results represent the mean ± SEM. **(D)** Real time RT-PCR was utilized to measure the levels of IFN-α, IFN-β and IFN-γ mRNA in tumors treated with or without alisertib. Results represent the mean ± SEM. **, $P<0.01$. **(E)** Real time RT-PCR was utilized to measure the levels of IFN-α, IFN-β and IFN-γ mRNA in CRC cell lines treated with or without alisertib. Results represent the mean ± SEM. *, $P<0.05$; **, $P<0.01$..

Figure S2. Deletion of Aurora-A by contributed to upregulation of STAT1, related to Figure 1.

(A-B) Real time RT-PCR was employed to measure the levels of Aurora-A (A) or STAT1 (B) mRNA. Results represent the mean ± SEM. The statistical significance was assessed by a paired t-test. *, $P<0.05$. **(C)** Immunoblotting was employed to measure the levels of Aurora-A, p-STAT1 and STAT1 in intestine tissues of $AURKA^{flox/+}$ and $AURKA^{flox/+};VillinCre^{+}$ mice. β-actin served as the control. **(D-E)** IF for STAT1 (green) and DAPI (blue) in intestine (D) and colon (E) sections from $AURKA^{f/+}$ or $AURKA^{f/+};VilCre^{+}$ mice.

Figure S3. Aberrant expression of STAT1 was required for alisertib resistance, related to Figure 1 and 2.

(A) Upon alisertib, the proliferation of cells was measured by MTT assay. Results

represent the mean ± SEM. **(B)** Annexin V and propidium iodide staining were analyzed by FACscan. **(C)** Real time RT-PCR was employed to measure the levels of the selected genes. Results represent the mean ± SEM. *, $P<0.05$; **, $P<0.01$. **(D-E)** Immunoblotting was employed to measure the levels of indicated protein. β-actin served as the control. **(F)** Upon alisertib, the proliferation of U251 cells infected with either GFP or STAT1 lentivirus was measured by MTT assay. **(G)** Immunoblotting was employed to measure the levels of indicated protein after knockdown of STAT1 in U251AR cells. **(H)** Upon alisertib, the proferation of U251AR cells transiently transfected with either control or an siSTAT1 was assessed by BrdU incorporation assay. Results represent the mean ± SEM. *, $P<0.05$; **, $P<0.01$. **(I)** Annexin V and propidium iodide staining were analyzed by FACscan. These results represent one of the experiments.

Figure S4. Inhibition of Aurora-A reduced Aurora-A and UHRF1 binding, related to Figure 3.

(A) The DNA methylation status was analyzed using Bisulfite sequencing analysis. **(B-C)** Cell lysates were harvested from U251 and U251AR (B), SW480 cells treated with or without alisertib (C), IP for either UHRF1 or Aurora-A, and immunoblotted with the indicated antibodies. Loading represents 5% of total cell lysate utilized for IP.

Figure S5. UHRF1 mediated DNMT1 degradation was critical for STAT1 expression, related to Figure 4.

(A-B) Immunoblotting was employed to measure the levels of indicated protein after knockdown of UHRF1 in HCT-8-7T (A) or HCT-8-9T (B) cells. **(C-D)** Immunoblotting was employed to measure the levels of indicated protein after overexpression of DNMT1 in HCT-8-7T (C) or HCT-8-9T (D) cells. **(E-F)** The proliferation of HCT-8-7T (E) and HCT-8-9T (F) cells transfected with control or DNMT1 vector was measured by MTT assay. Results represent the mean ± SEM.

Supplementary Figure 1

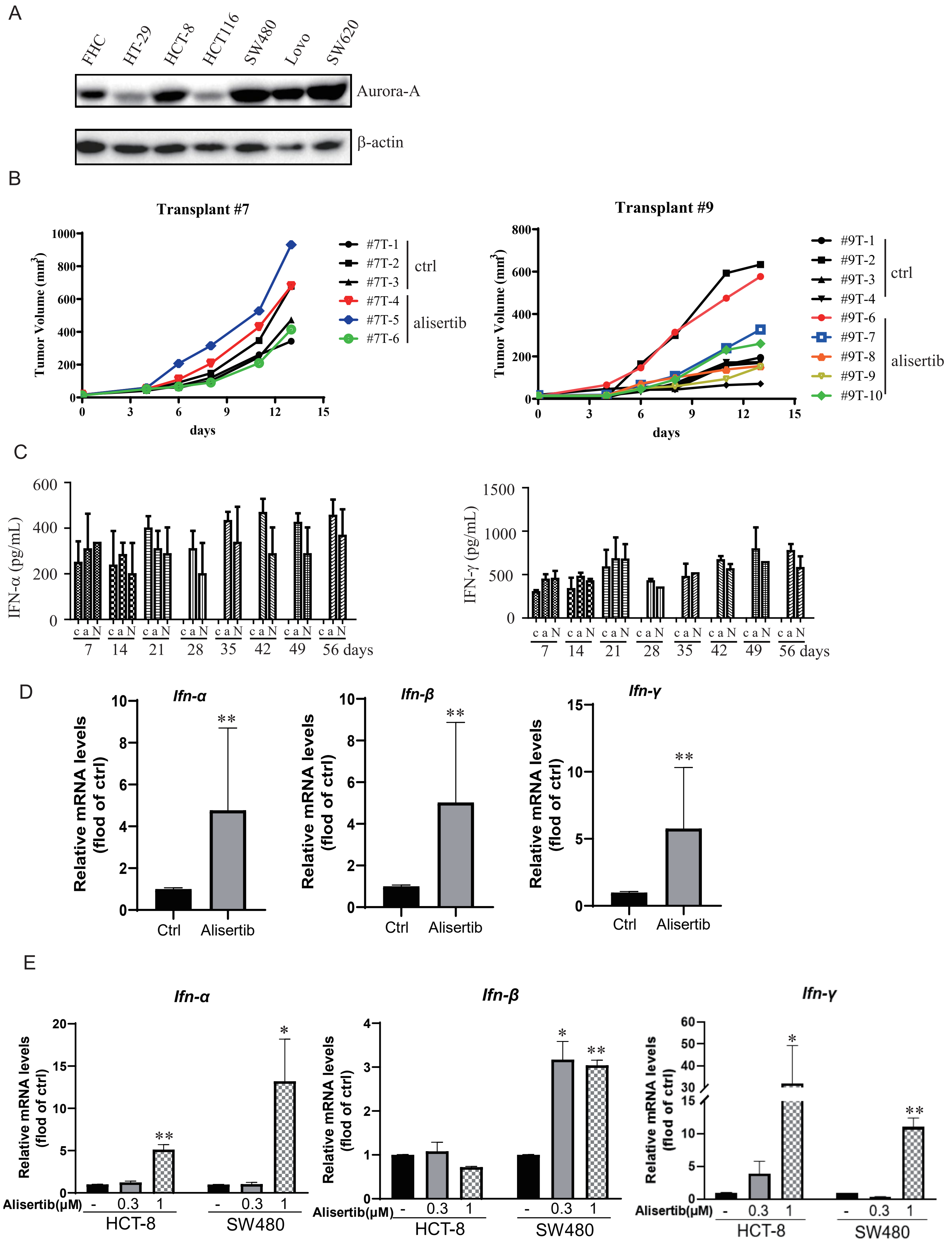

Supplementary Figure 2

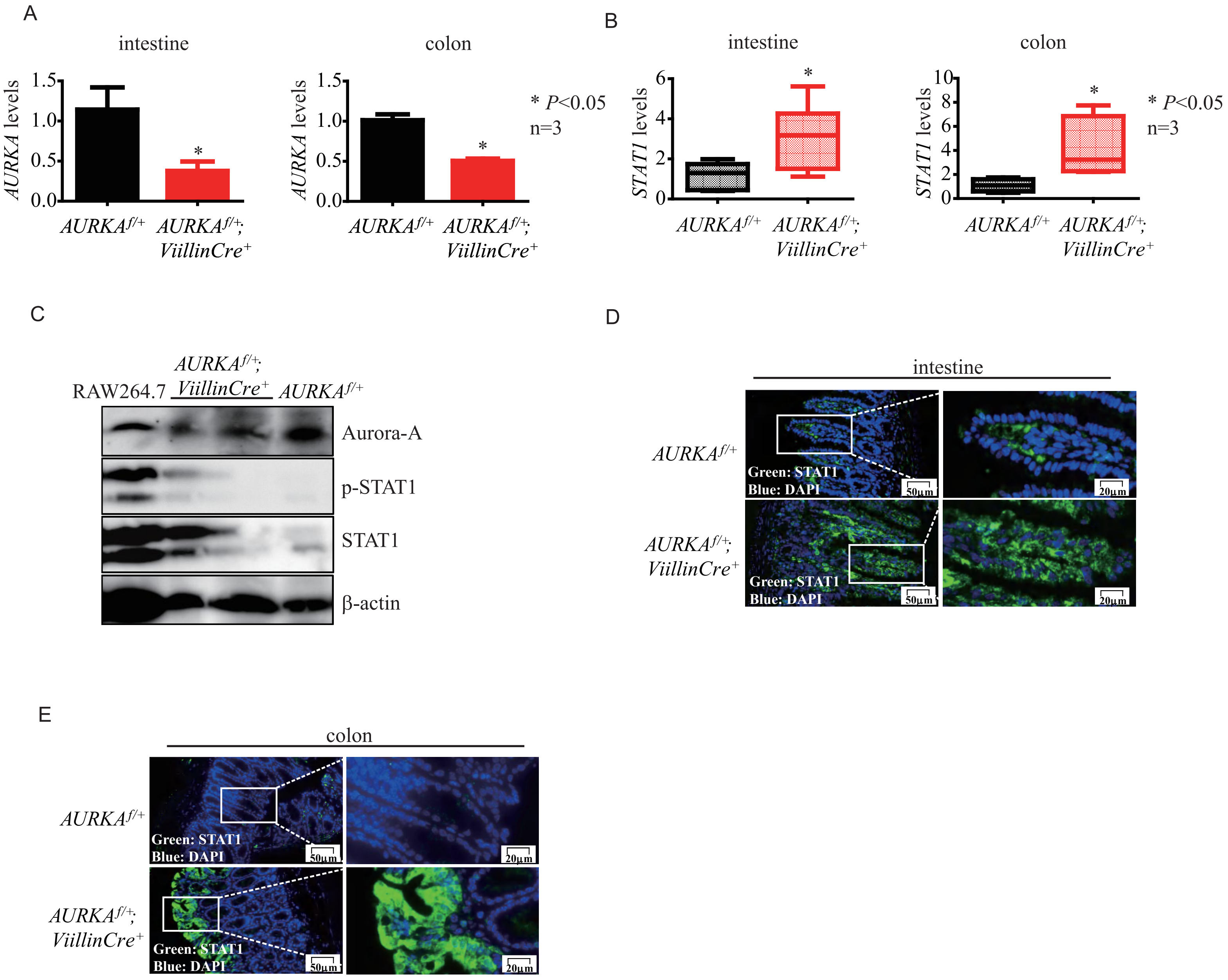

Supplementary Figure 3

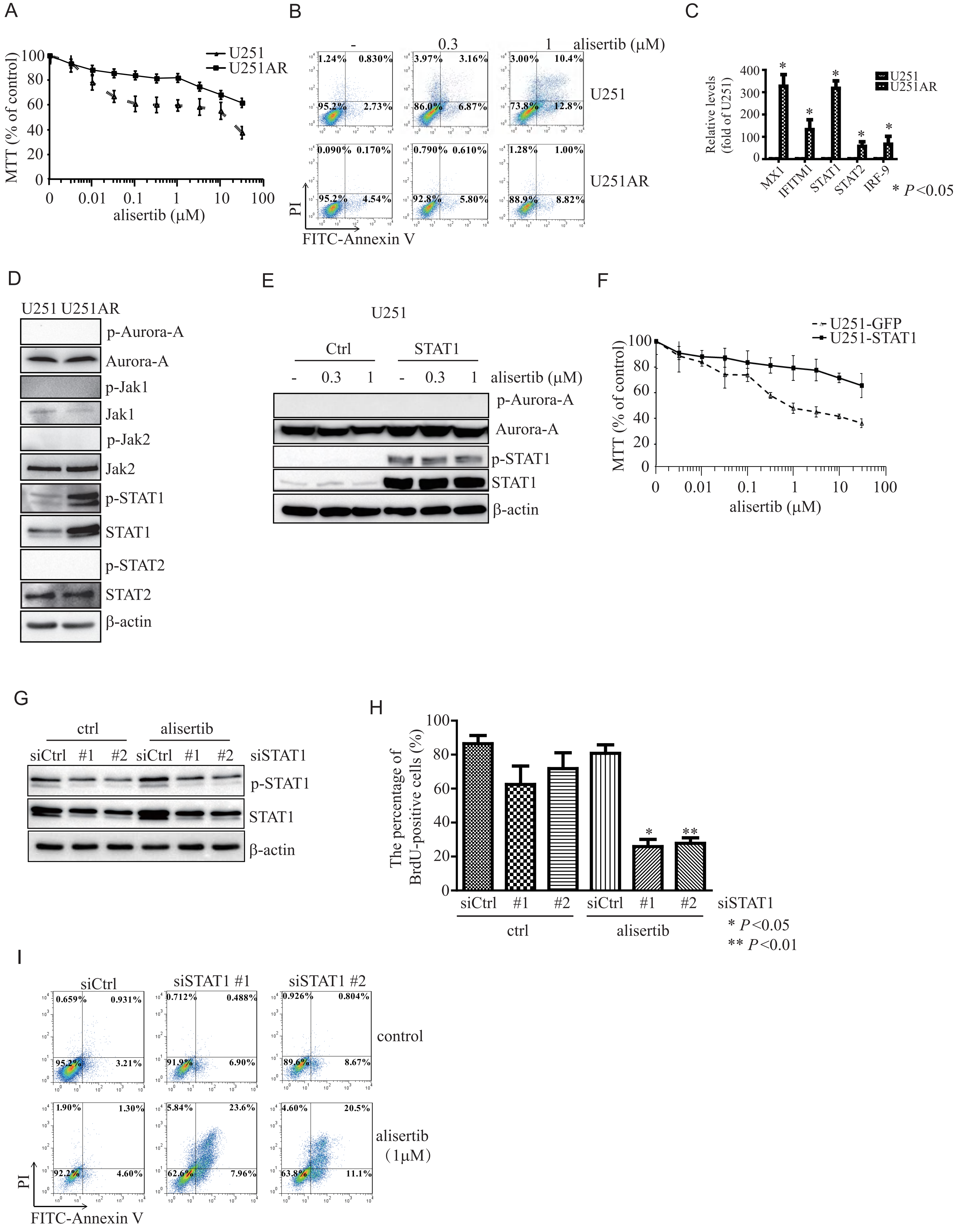

Supplementary Figure 4

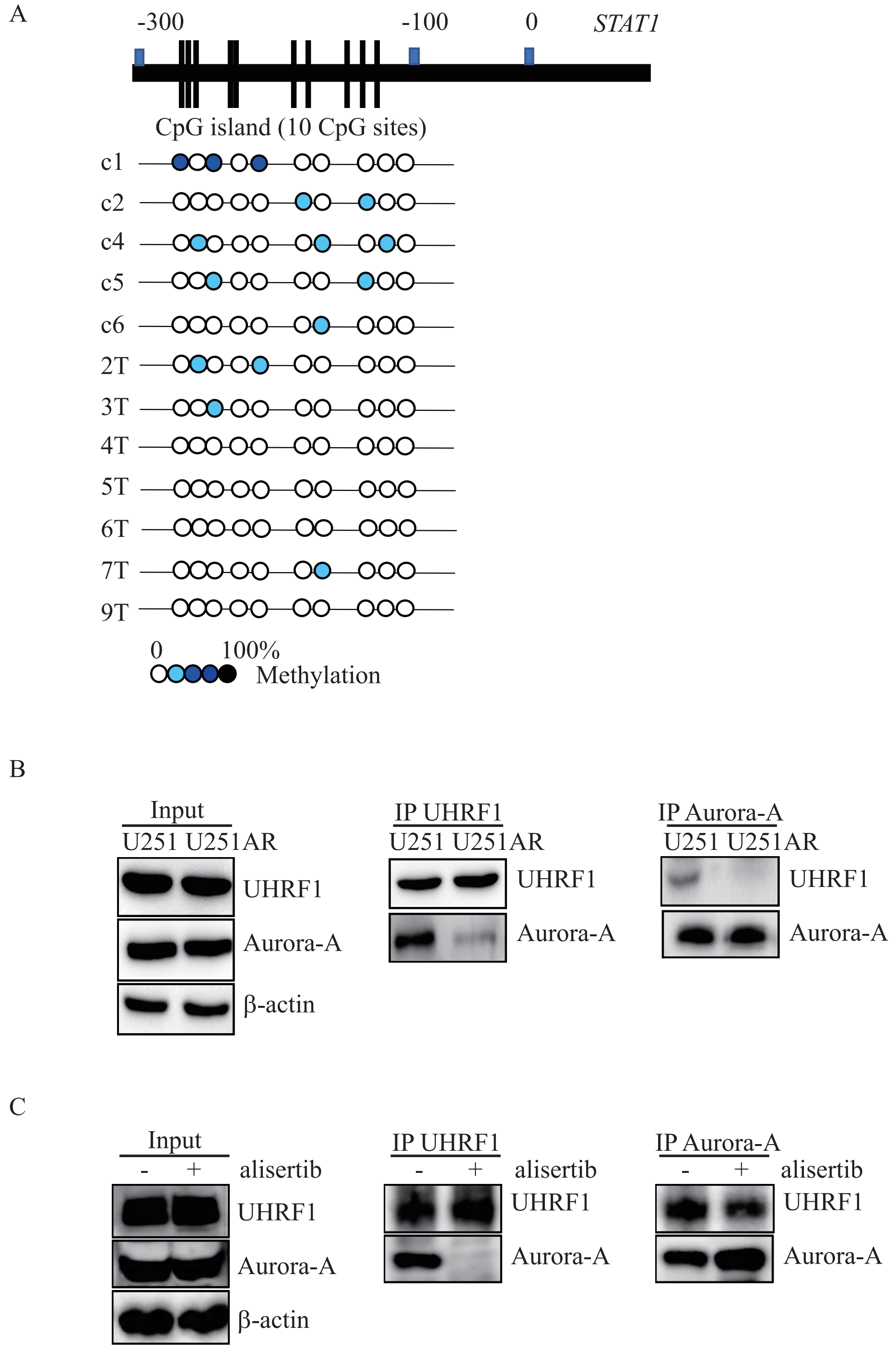

Supplementary Figure 5

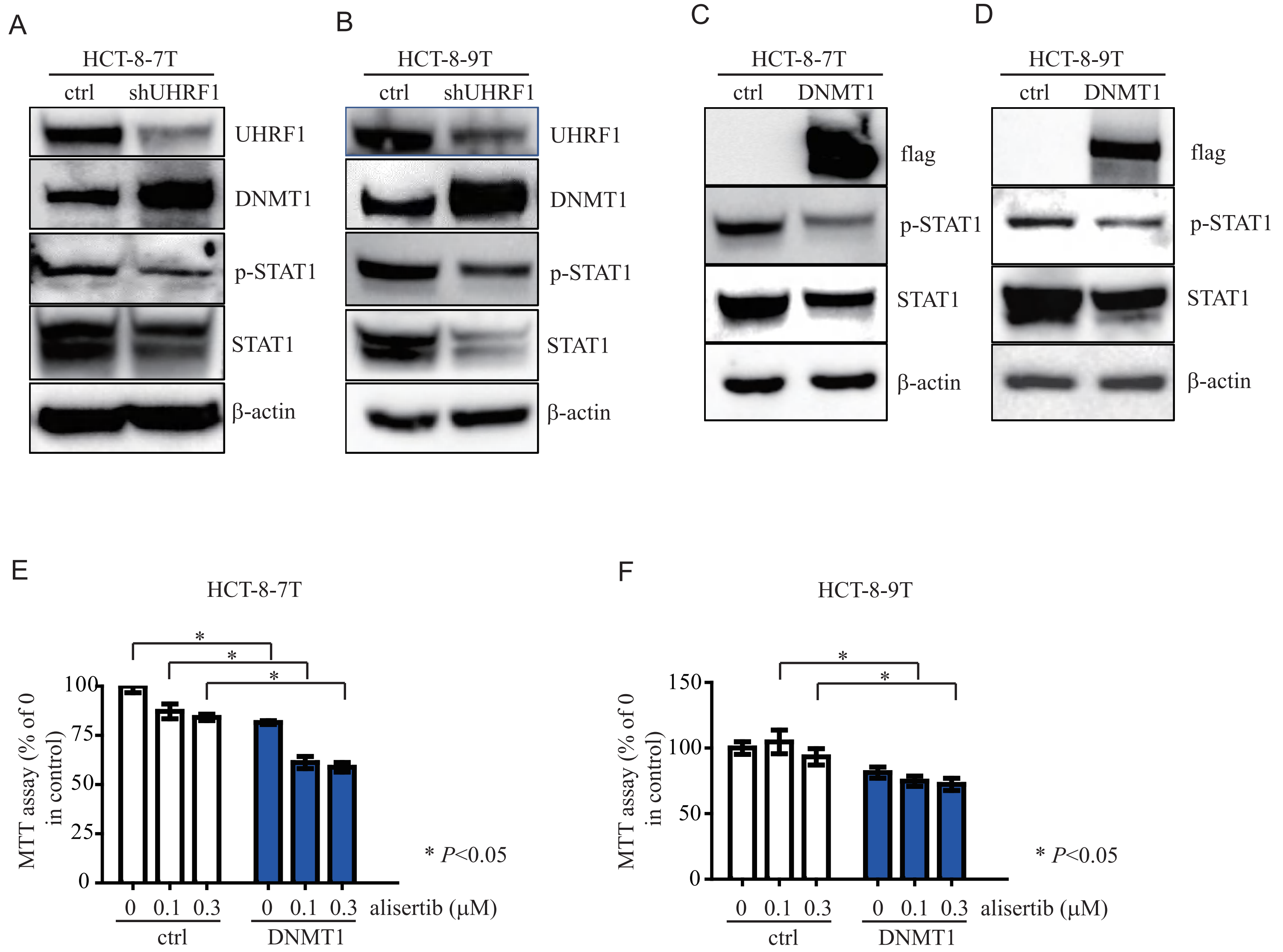

Supplement: Supplementary file 1 — Supplementary figures and tables. [file ijmsv18p3437s1.pdf]
